# Study about the effect of cellulose nanocrystals on a polyacrylate miniemulsion

**DOI:** 10.1039/d4ra06614f

**Published:** 2025-03-06

**Authors:** Zeping Wang, Lionel O'Young, Sajid Mahmood, George Zheng Chen, Yitao Zheng, Binjie Hu

**Affiliations:** a Green Chemicals & Energy Process Development Laboratory, China Beacons Institute, University of Nottingham Ningbo China 199 Taikang East Road Ningbo 315000 China; b Low Dimensional Materials Research Center, Khazar University Baku AZ1096 Azerbaijan; c Department of Chemical and Environmental Engineering, Advanced Materials Research Group, Faculty of Engineering, The University of Nottingham Nottingham NG7 2RD UK; d Department of Chemical & Environmental Engineering, University of Nottingham Ningbo China 199 Taikang East Road Ningbo 315000 China Binjie.HU@nottingham.edu.cn

## Abstract

Cellulose nanocrystals (CNC) are widely used due to their biodegradability, high strength, large surface area, and functional versatility. This study investigates the interaction between CNC and acrylate emulsions, which mainly focuses on their impact on emulsion characteristics, polymerization behaviour, and storage stability. CNC was incorporated into an acrylate miniemulsion system at varying concentrations, followed by the systematic study of its effects on particle size, interfacial tension, zeta potential, yield, and viscosity. The morphology of CNC-acrylate systems was analysed using infrared spectroscopy and scanning electron microscopy (SEM). The results demonstrated that CNC effectively co-stabilized acrylate miniemulsions and enhanced their stability before polymerization. Although CNC did not directly participate in polymerization or affect yield or reaction rates, it slowed the diffusion of free radicals. However, CNC concentrations higher than 1 wt% negatively impacted post-polymerization storage stability and caused aggregation of droplets. These findings reveal the dual role of CNC as both a stabilizing and aggregating agent, offering new insights into its potential for the design of advanced polymer systems.

## Abstract 2

This study investigates the interaction between CNC and acrylate emulsions, which mainly focuses on their impact on emulsion characteristics, polymerization behaviour, and storage stability.

## Graphical abstract

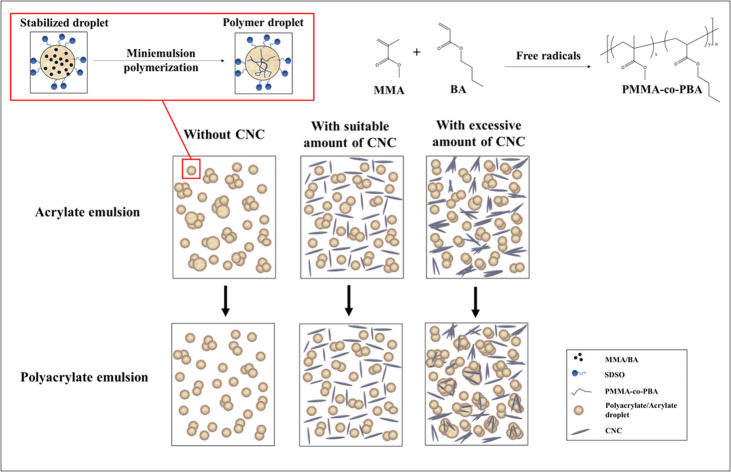

## 1. Introduction

In the coating industry, there is a potential to transform through sustainability, improved performance, and multifunctionality. Waterborne coatings are increasingly favoured due to their reduced environmental impact in lower volatile organic compound (VOC) emissions an alignment with global sustainability goals and regulatory requirements.^1^ These coatings utilize water as the primary solvent, thus offering an eco-friendly solution. However, challenges like suboptimal mechanical strength, stability in waterborne systems have necessitated the development of innovative additives to improve performance.^2^

To solve those challenges, seeking more novel potential additive materials could be a solution. Cellulose as the most abundant biopolymer on earth, has drawn more attention recently on its various additive function due to its ease of altering, tailoring and manufacture.^3,4^ Cellulose nanocrystal (CNC), a rod-shaped nanomaterial extracted from cellulose microfibrils, has gained significant attention for their unique properties like high crystallinity and strong mechanical properties, and diverse applications.^5^ Compared with cellulose nanofiber (CNF), CNC is shorter in length and has a higher ratio of crystalline structure, exhibiting high specific strength, modulus, large surface area, gas impermeability, and unique liquid crystalline properties.^6,7^ In addition, CNC also shares advantages of cellulose, which are biodegradable, biocompatible, renewable and environmentally friendly.^8^ Attracted by these characteristics, CNC is widely applied in nanocomposites films and membranes for food packaging,^9^ conductive materials,^10^ water treatment,^11^ biomedical use,^12^ and waterborne coatings,^13–16^ especially. Moreover, the enhancement of CNC on film strength, water resistance, and environmental sustainability was also proven inside a waterborne polyacrylate system in architectural, automotive, and anticorrosive coatings.^17–19^

Despite these developments on CNC, previous research mainly focused on the applications of CNC, not much systematic research has been carried out. Especially the mechanisms and behaviour of CNC within coatings is yet understood. To address this gap, this study systematically investigates the effects of CNCs within a waterborne polyacrylate coating, providing deeper insights into their role and interactions between CNC and polymers within the coating.

## 2. Experimental methods and materials

### 2.1. Materials

Methyl methacrylate (MMA, CP), sodium dodecyl sulfonate (SDSO, CP), l-ascorbic acid (AAc, AR) and hydrogen peroxide (H_2_O_2_, AR) were supplied by Sinopharm Chemical Reagent Co., Ltd. Hexadecane (HD, 98%) and *n*-butyl acrylate (BA, 99%) were offered by Aladdin Industrial Inc. Cellulose nanocrystal (CNC) was purchased from ScienceK Co., Ltd. Ultra-pure water (18.2 MΩ cm) was obtained from a ultrapure water system (Milli-Q® IQ 7000).

### 2.2. Preparation of waterborne acrylate coating

#### 2.2.1. Preparation of oil phase

The oil phase was mixed with HD (5.0 wt%), MMA (47.5 wt%) and BA (47.5 wt%) under magnetic mixing at 200 rpm, where HD was used for the prevention of Ostwald ripening.

#### 2.2.2. Preparation of water phase

The water phase was formed with water, SDSO (30 mM) and CNC (0, 0.1, 0.5, 1, 1.5 and 2 wt%) under magnetic stirring for 30 minutes.

#### 2.2.3. Homogenization of oil phase and water phase

The oil phase and water phase were homogenized in a sonicator (Xinzhi Scientz II) for 6 minutes (1 second on and 1 second off) with oil-in-water (O/W) volume ratio of 3 : 7 at 40 °C and 285 W.

### 2.3. Miniemulsion polymerization

After homogenization of oil phase and water phase, the mixture was loaded into a 250 mL four-necked flask equipped with a stirrer, a reflux condenser, a thermometer, and a nitrogen inlet. The temperature was controlled at 40 °C with a water bath. After stirring at 200 rpm and nitrogen bubbling for 1.5 h to remove oxygen, H_2_O_2_/AAc solution (0.1 mol% of the oil phase) was introduced to initiate the polymerization.

### 2.4. Characterization techniques

#### 2.4.1. Droplet size

The droplet sizes and its size distribution were measured with a laser diffraction method (Mastersizer 3000, Malvern Inc).

#### 2.4.2. Interfacial tension

The interfacial tension at the oil/water interface was measured using the Du Noüy sheet method (BZY-2 tensiometer, Hengping Instruments).

#### 2.4.3. Zeta potential

Zeta potentials of the acrylate miniemulsion were measured with a trace laser doppler electrophoresis method (ZetasizerNano ZS, Malvern Inc.) at room temperature. The sample was diluted one hundred times with water and pH of each sample was controlled at 5 to prevent the pH interference. For each sample, the measurement was repeated for three times.

#### 2.4.4. SEM

The morphology of polyacrylate samples with or without CNC was studied by the scanning electron microscopy (SEM) (RIGMA/VP, Carl Zeiss Microscopy Ltd) under a 3 kV accelerating voltage. The polyacrylate emulsions were diluted one thousand times, dropped on a silicon slice, dried in air and placed at a platform for observation.

#### 2.4.5. Yield

The yield of PMMA-*co*-PBA was calculated gravimetrically due to the volatile nature of monomers and hydrophobes. Samples were simultaneously taken at regular intervals and stored in an ice bath to terminate reaction. After that, samples were placed in a fume cupboard to evaporate the monomer and hydrophobe residue. Then samples were baked in a vacuum oven at 120 °C for 4 h to remove moisture. The yield of polymers could be obtained from the mass ratio of polymer and monomer.

#### 2.4.6. Viscosity

Viscosities of miniemulsion after polymerization were measured using the rotational rheometer with a plate-and-plate system (Kinexus Pro+, Malvern Inc), where the diameter of the upper plate is 40 mm and the gap between the upper and lower plates is 1.0 mm. Moreover, the measurement was conducted at a shear rate ranging from 0.1 s^−1^ to 1000 s^−1^ at room temperature (25 °C).

#### 2.4.7. FTIR

FTIR spectra of polyacrylate samples with or without CNC was collected from the Bruker vertex 70 instrument.

## 3. Results and discussion

### 3.1. Effect of the CNC on the o/w interface of acrylate miniemulsion

**Fig. 1:**
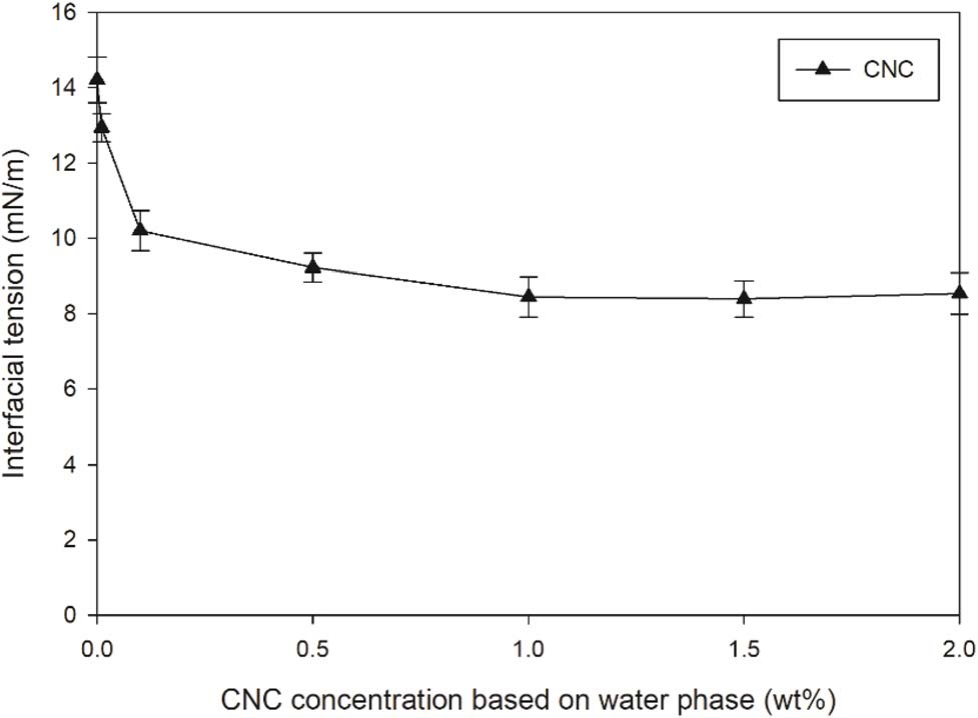
Variation in interfacial tension at different CNC contents in water phase (without SDSO).

The polyacrylate emulsion was composed of an oil phase, a water phase, and surfactants, which was proven to be an effective coating formula in our previous studies.^20,21^ Initially, to study the effect of CNC on the o/w interface, the interfacial tension between the oil and water phase at different concentrations of CNC without SDSO was measured and presented in Fig. 1. The interfacial tension remarkably decreased from 14.2 mN m^−1^ to 10.2 mN m^−1^ after introducing 0.1 wt% CNC. The value further decreased to 9.2 mN m^−1^ as the CNC content increased to 1 wt%, then remained relatively constant with further increases in CNC content up to 2 wt%. This validated that the CNC could effectively reduce the interfacial tension between the oil and water phase. However, the mixture of oil and water phases after ultrasonication could not achieve a homogeneous emulsion without SDSO. In other words, the CNC as received could not fully stabilize the o/w interface. Therefore, SDSO was introduced as the main stabilizer into this formulation. 30 mM was the cost-effective concentration for SDSO in an emulsion.^22^

**Fig. 2:**
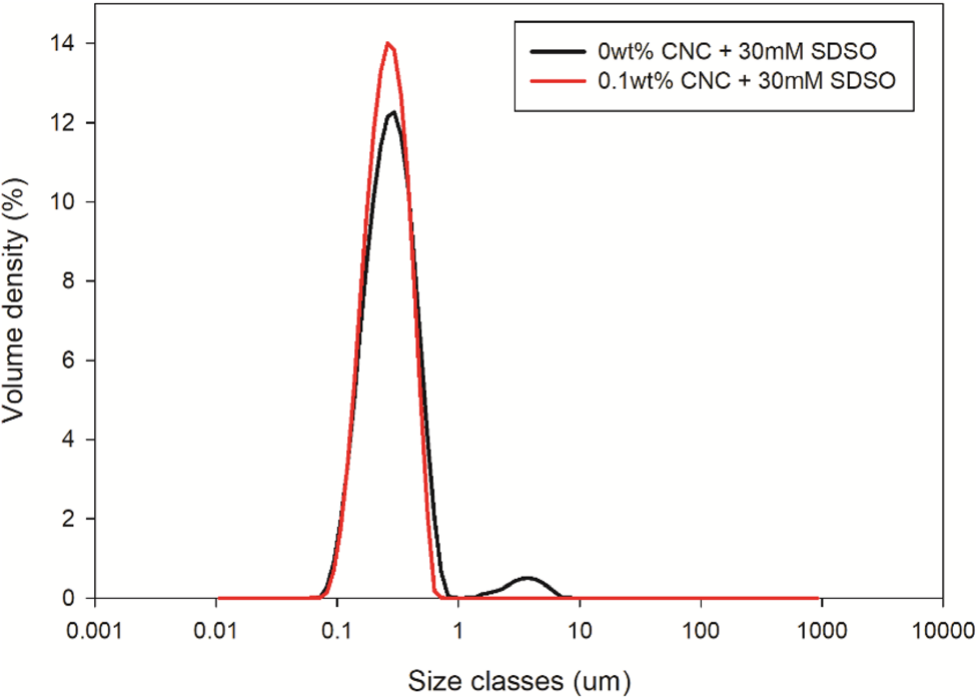
Variation in initial droplet size distribution with or without 0.1wt% CNC in water phase (30 mM SDSO).

After adding 30 mM SDSO, the emulsion was supposed to be homogenous without CNC. However, there was still a small portion of the emulsion remained unstable. As shown in Fig. 2, two peaks were observed in the size distribution plot of emulsion without CNC, where the main peak distributed in the range of 0.1–1 μm, and the other small peak located between 1–10 μm. The main peak represented the stable emulsion, while the smaller peak could be ascribed to the flocculation or partial coalescence of a small quantity of oil droplets.^23,24^ In comparison, the emulsion with 0.1 wt% CNC presented only a single, tight peak in the size distribution plot, which indicated that the introduced CNC could effectively prevent flocculation or partial coalescence of oil droplet. In other words, CNC could act as a co-stabilizer in this coating formula. Therefore, the Sauter mean diameter (*D*_3,2_) of oil droplet decreased from 248 nm to 235 nm after introducing 0.1 wt% CNC.

**Fig. 3:**
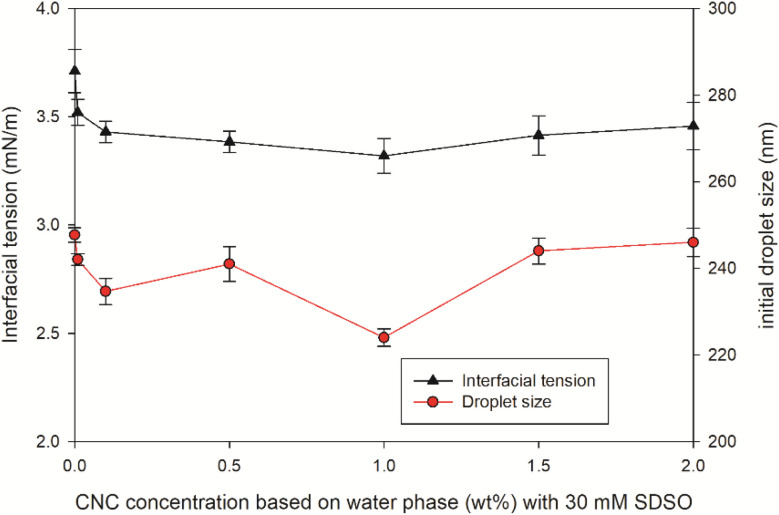
Effect of CNC content on the initial droplet size and interfacial tension between oil and water phase (30 mM SDSO).

To prove the co-stabilization effect of CNC, the interfacial tension and droplet size at different CNC contents with 30 mM SDSO was measured and presented in Fig. 3. The interfacial tension reduced from 3.7 mN m^−1^ to 3.4 mN m^−1^ after the CNC content reaching 0.1 wt%. With increasing the CNC content to 1 wt%, the interfacial tension further reduced to 3.3 mN m^−1^, despite that the reduction was limited, Similar reductions in the interfacial tension were recorded by Hu, *et al.*,^25^ which was attributed to the intermediate wettability of CNC. The interfacial tension and particle size were positively correlated.^26^ This was proven by the change of initial droplet size in Fig. 3. More particles could nucleate at lower interfacial tension.^27^ For a fixed volume of oil phase, more particles indicated the smaller size in singe droplet.

CNC was reported as a Pickering agent to stabilize the oil-water interface in emulsions due to intermediate wettability and nanometric size.^28–32^ Pickering emulsions were also known as particle-stabilized emulsions. Like other Pickering agents, CNC could prevent coalescence and sedimentation by forming a tightly packed layer.^33^ Although there was still a debate on whether particles could also decrease the interfacial tension like surfactants.^34^ Some literature insisted claiming that they found the reduction of interfacial tension with particles as stabilizers.^35–39^

**Fig. 4:**
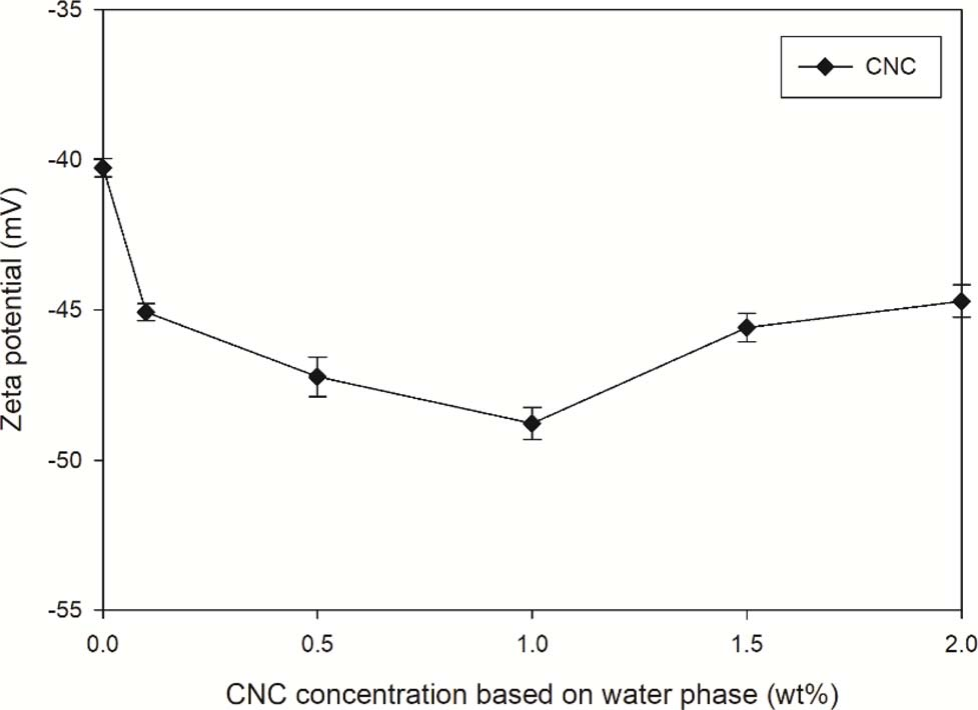
Effect of CNC content on the zeta potential (30 mM SDSO).

To further validate the co-stabilization effect, zeta potential of each sample was measured and presented in Fig. 4. Empirically, the emulsion was considered as a stable system if the potential values higher than +30 mV or lower than −30 mV, which indicated that particles repelled each other and form a stable dispersion.^40^ Moreover, a higher absolute value of zeta potential referred to a higher stable state of colloidal systems.^41^ In this way, all the samples with or without CNC were stable. After introducing 0.1 wt% CNC, the absolute value of zeta potential increased from 40.2 to 45.1 mV, indicating the stability enhancement. This is another evidence of CNC to be the co-stabilizer.

### 3.2. Effect of the CNC on the acrylate miniemulsion polymerization

#### 3.2.1. Effect of CNC on miniemulsion polymerization

**Fig. 5:**
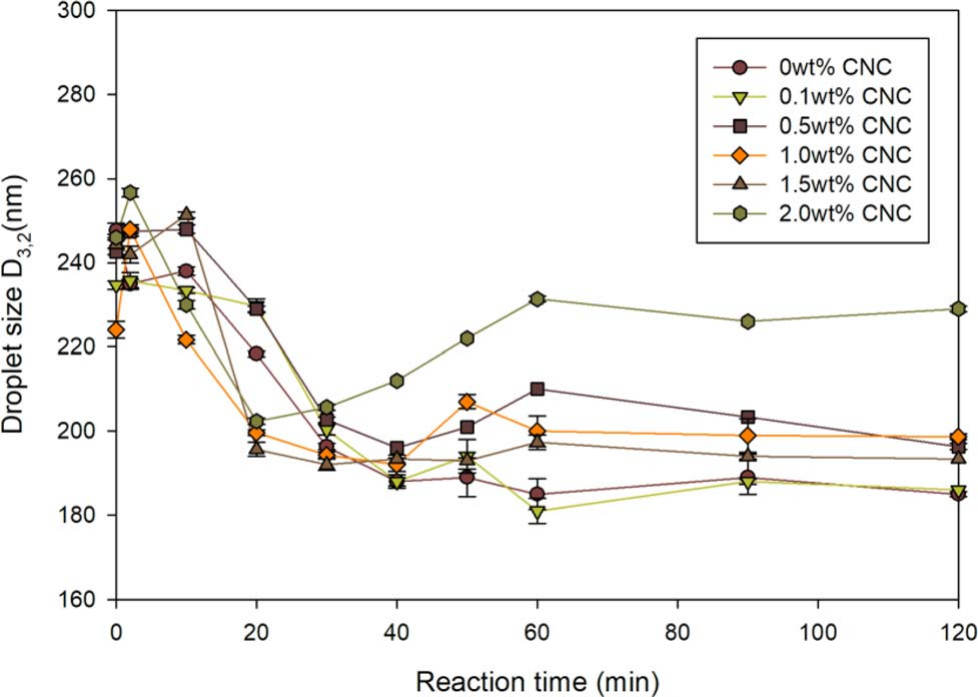
Droplet size of each sample during the reaction as a function of CNC content.

**Fig. 6:**
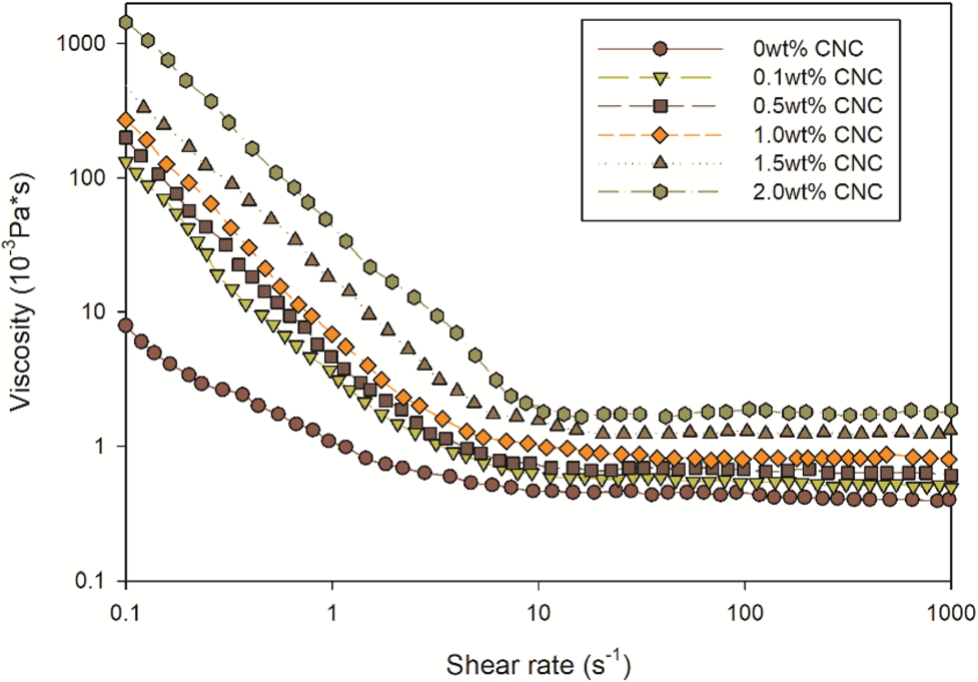
Variation in viscosity *versus* shear rate as a function of CNC content.

After preparing the miniemulsion, the effect of CNC on droplet size and reaction during miniemulsion polymerization was investigated. As shown in Fig. 5, *D*_3,2_ of various samples with different CNC contents varied over time. After introducing initiators, *D*_3,2_ of all samples exhibited a slight rise due to temporary instability. Unlike emulsion polymerization, droplet nucleation was the predominant nucleation mode.^42^ In other words, droplets nucleated and formed polymer particles. Except 2 wt% CNC sample, the reduction of *D*_3,2_ during the first hour could be attributed to the formation of polymer particles. Moreover, the relatively constant *D*_3,2_ in the second hour indicated the termination of miniemulsion polymerization. However, among these samples, the size change of 2 wt% CNC sample differed. *D*_3,2_ of 2 wt% CNC sample presented an increasing trend at first 20 min, which could be ascribed to the depletion flocculation of emulsion.^43^ Depletion flocculation occurred in oil-in-water emulsions when the concentration of non-adsorbed polysaccharide exceeded a certain level. In this study, CNC was a type of polysaccharide. When the CNC content was higher than 2 wt%, instability occurred and caused the aggregation or coagulation of droplets. To prove this, the viscosity of various samples at different shear rates was measured and displayed in Fig. 6. All the samples presented the shear thinning behaviour, particularly at 2 wt%. The viscosity at 0.1 s^−1^ was 100 times higher than the one of the sample without CNC, which indicated aggregation or coagulation of droplets breaking with increasing shear rate.^44^

**Fig. 7:**
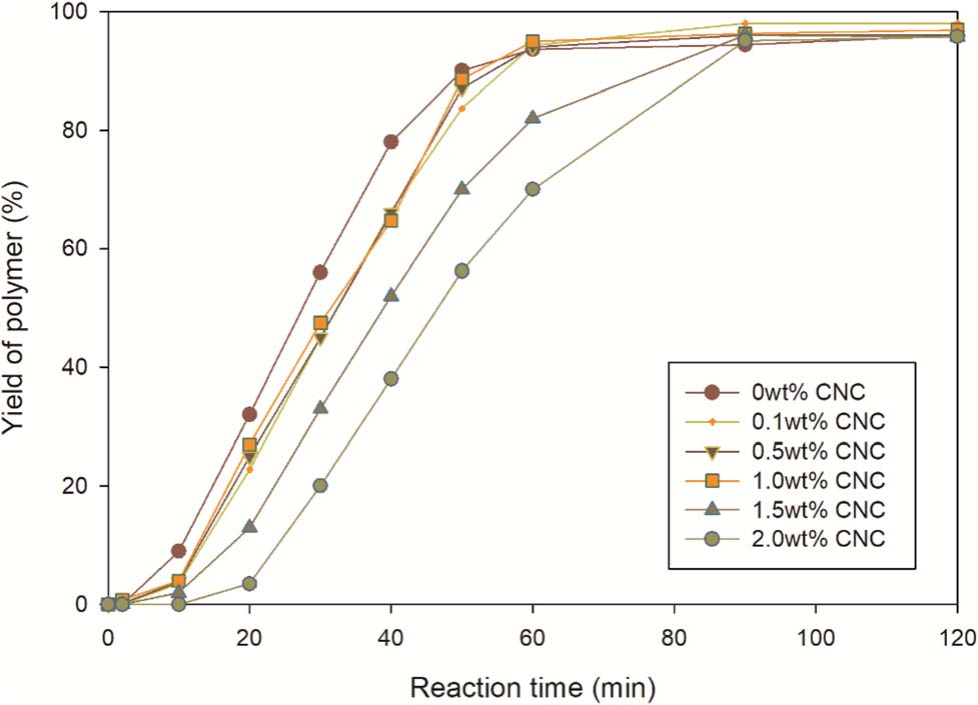
Variation in yield as a function of CNC content.

In addition to the droplet size, the yield of miniemulsion polymerization at different CNC contents was calculated and displayed in Fig. 7. The yield of each sample was similar, approximately 96% at the end of the experiment. Most of samples achieved the highest yield within first hour, except for the 1.5 wt% and 2 wt% CNC samples. The difference in termination time might be affected by the rate of miniemulsion polymerization *R*_p_ and diffusion coefficient *D* of initiator from water phase to monomer droplet. Bechthold and Landfester^45^ proposed an equation to calculate miniemulsion polymerization rate *R*_p_ (eqn (1)).1
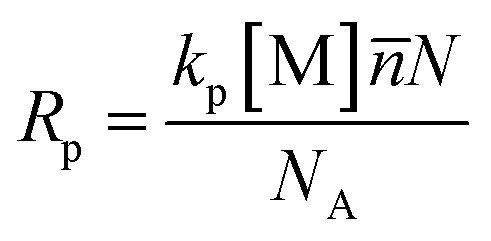
where *k*_p_ was the reaction rate constant, [M] was the monomer concentration, *n̄* was the average effective radical number per droplet, *N* was the number of droplets per liter of water phase, and *N*_A_ was the Avogadro's number.

In this study, the reaction temperature, monomer and initiator amount concentration were fixed, so the value of *k*_p_, [M] and *n̄* remained constant. As for the number of droplets per liter of water phase *N*, it could be calculated with eqn (2).2
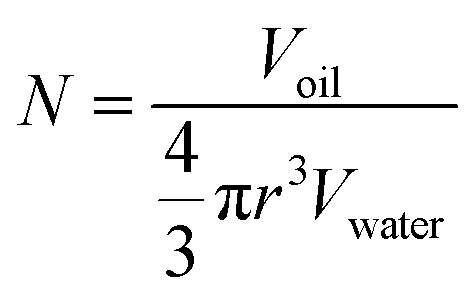
where *r* was droplet radius, *V*_oil_ and *V*_water_ represented the volumes of the oil phase and water phase, respectively. The volume ratio of the oil and water phase was identical in this study. The only difference was the radius of droplet, which ranged from 225 to 250 nm, still within the same size range. Therefore, the curves of linear increasing phase were parallel to each other and *R*_p_ of each sample was similar.

However, the reaction initiation point was different, which could be influenced by the diffusion of free radicals from initiator to monomer. The Stokes–Einstein equation in rotational diffusion^46^ was given as listed,3
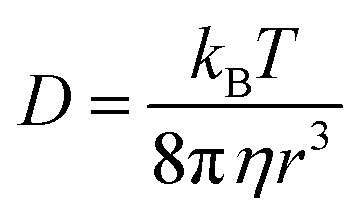
where *D* was diffusion coefficient, *k*_B_ was the Boltzmann's constant, *T* was absolute temperature, *η* was dynamic viscosity and *r* was radius of the spherical particle. In this equation, diffusion coefficient *D* was inversely related to viscosity *η* and the radius *r* of the moving particles. As shown in Fig. 6, the viscosity increased with the increment of CNC loading. Therefore, it would take longer time for free radicals to transport into samples with higher CNC content. And after that, once the free radicals reached the reaction site and miniemulsion polymerization initiated, all the samples presented the similar reaction rate.

#### 3.2.2. Effect of CNC on interactions with PMMA-*co*-PBA inside the polyacrylate coating

**Fig. 8:**
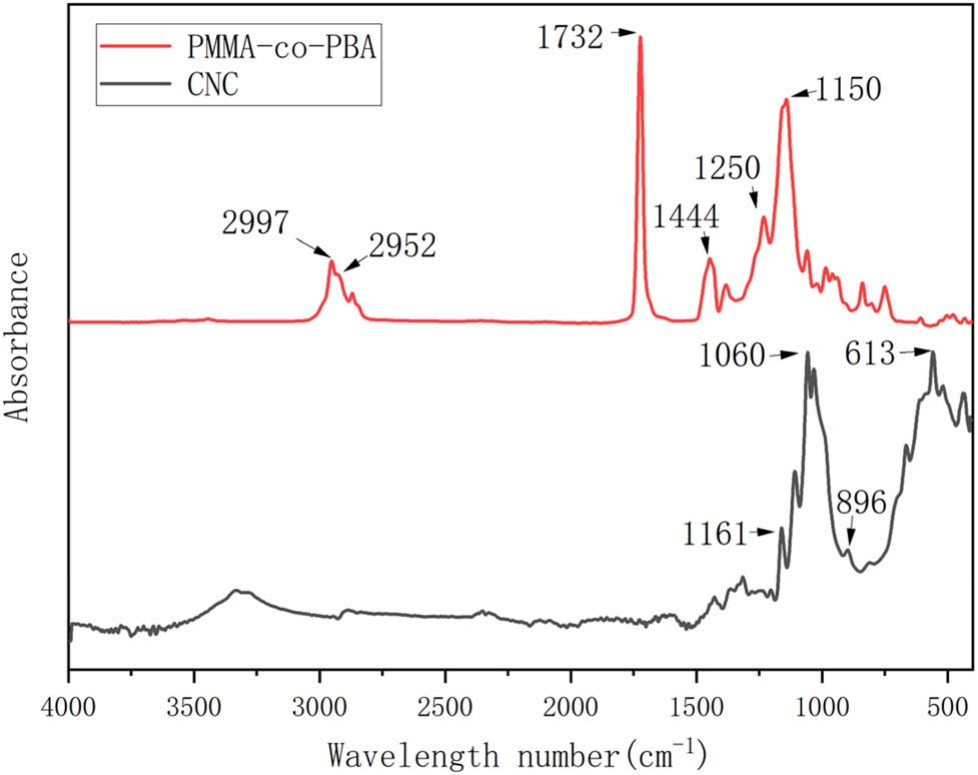
FTIR spectra for PMMA-*co*-PBA and CNC.

In addition to the effect of CNC on miniemulsion polymerization, interactions of CNC between PMMA-*co*-PBA were another area of investigation. Motivated by this, CNC as received and polyacrylate samples with different CNC contents were analysed using FTIR. Firstly, the FTIR of CNC and PMMA-*co*-PBA were measured and presented in Fig. 8. For PMMA, peaks at 2997 cm^−1^, 2952 cm^−1^ and 1444 cm^−1^ were assigned to the C–H stretching vibrations of –CH_3_ and –CH_2_- groups, and the C–H bending vibration of –CH_3_ and –CH_2_- groups, respectively.^47,48^ Peaks at 1732 cm^−1^, 1250 cm^−1^ and 1150 cm^−1^ represented the stretching vibrations of C=O and C–O in ester group, respectively, validating the existence of PMMA-*co*-PBA.^49,50^ For CNC, the peak at 613 cm^−1^ was caused by the stretching vibration of aromatic –CH.^51^ The peak at 896 cm^−1^ could be the characteristics of the C–O–C symmetric stretching vibration of β-glycosidic linkages.^52^ Additionally, peaks at 1161 cm^−1^ and 1060 cm^−1^ corresponded to the stretching vibration of C–O–C in the pyranose ring and the bending vibration of C–O–C in β-glycosidic linkages, respectively.^53–56^ For the ease of comparation, these characteristic peaks were summarized and listed in Table 1.

**Table 1:** Peak assignment for CNC-incorporated PMMA-*co*-PBA

Peak location (cm^−1^)	Origin of peak	Reference
2997	C–H stretching vibration of –CH_3_ groups	47
2952	C–H stretching vibration of –CH_2_- groups	47
2899	C–H asymmetric stretching vibration in glucose units	56 and 57
2845	C–H symmetric stretching vibration in glucose units	57
1732	C=O stretching vibration	49 and 50
1444	C–H bending vibration of –CH_3_ and –CH_2_- groups	48
1250, 1150	C–O stretching vibrations of ester groups	49 and 50
1161	C(O)–O stretching vibration in pyranose ring	53–55
1060	C–O–C bending vibration of β-glycosidic linkages	56
896	C–O–C symmetric stretching vibration of β-glycosidic linkages	52
613	Aromatic –CH stretching vibration	51

**Fig. 9:**
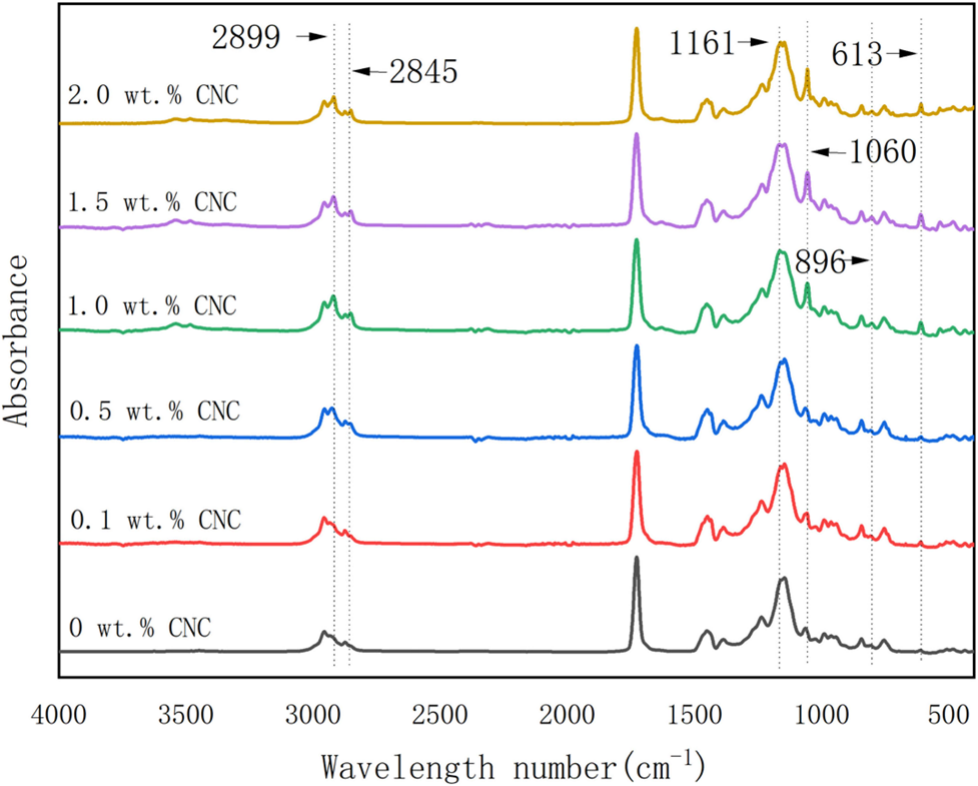
FTIR spectra for polyacrylate samples containing different CNC contents.

As shown in Fig. 9, FTIR spectra of polyacrylate samples with different CNC contents were presented. In the spectra, the peaks at 1161, 1060, 896, and 613 cm^−1^ became more apparent when CNCs content exceeded 0.5 wt%, which indicated the successful addition of CNC. Moreover, peaks at 2899 cm^−1^ and 2845 cm^−1^ were also observed in the samples with higher CNC contents (>0.5 wt%). These two peaks were categorized to C–H asymmetric and symmetric stretching vibration in glucose units, respectively.^56,57^ Meanwhile, CNC contained glucose units. In other words, this was another evidence that CNC was successfully incorporated into the polyacrylate samples. However, no other new peaks were observed, which indicated that no new substance formed. Therefore, CNC was estimated to exist between PMMA-*co*-PBA without attending the reaction.

**Fig. 10:**
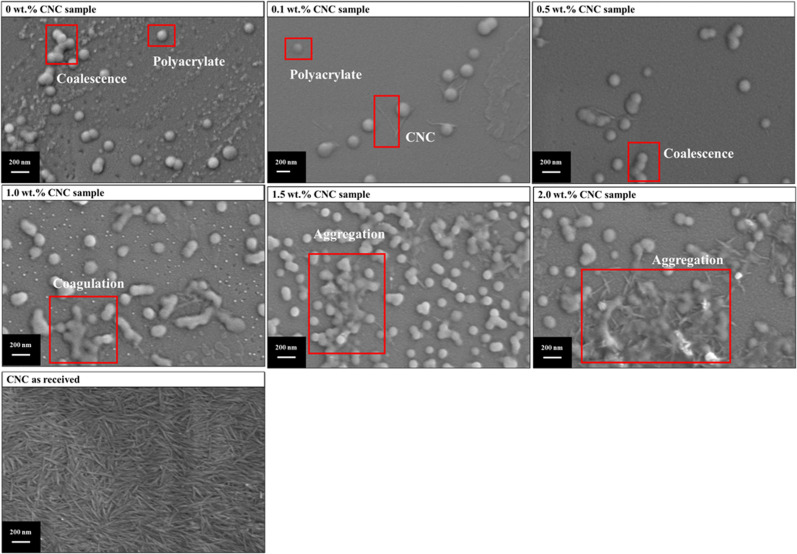
SEM for polyacrylate samples containing different CNC contents and CNC as received.

To further evaluate the interaction between CNC and PMMA-*co*-PBA, the morphology of polyacrylate samples with or without CNC and CNC as received was investigated with SEM, which was shown in Fig. 10. For the polyacrylate sample without CNC, both the single droplet and coalesced droplets could be observed. After adding 0.1 wt% CNC, the polyacrylate sample was dispersed, and CNC located near the droplet. Similar phenomenon was observed in the 0.5 wt% sample, while the coalescence of droplets was more severe. With further increasing the CNC content to 1 wt%, the coagulation happened. Then the aggregation was found in 1.5 wt% and 2 wt% samples, where the aggregation of 2 wt% is relatively harder.

The interaction between CNC and PMMA-*co*-PBA was further evaluated using SEM to analyse the morphology of polyacrylate samples with varying CNC concentration (Fig. 10). Fig. 10g confirmed the rod-like shape of CNC. In the absence of CNC. In the absence of CNC (Fig. 10a), the polyacrylate sample exhibited the coexist of individual droplets and droplets coalescence, which suggests the instability in such system. Adding 0.1 wt% CNC (Fig. 10b) resulted in noticeable uniform dispersion within the polymer matrix, with CNC particles predominantly localized near the droplets. This suggested that presence of CNC at lower concentrations could prevent droplets coalescence even. This further suggests that CNC could acted as a stabilizing agent in emulsion formation. However, further increase CNC concentration to 0.5 wt%, the droplet coalescence became more pronounced. As the CNC content increased to 1 wt%, coagulation was observed. At higher CNC concentrations (1.5 wt% and 2 wt%), significant aggregation occurred, with the most severe aggregation seen in the 2 wt% sample. This phenomenon the function of CNC as co stabiliser is limited at lower concentration. At higher concentration, CNC particle–particle interactions become dominant to induce droplets coalescence hence jeopardise stability of emulsion.^58^

**Fig. 11:**
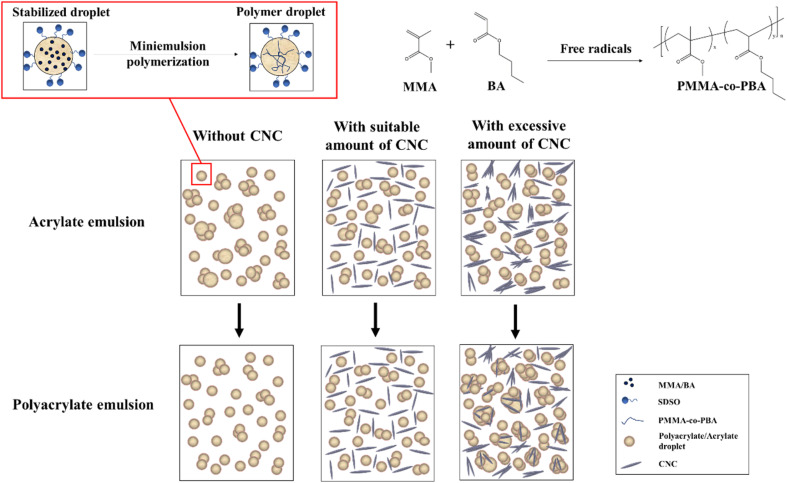
Conceptual diagram of the interaction between CNC and PMMA-*co*-PBA.

These results highlighted the dual role of CNC as both a stabilizing and aggregating agent, depending on its concentration. As shown in Fig. 11, the interaction between CNC and PMMA-*co*-PBA was illustrated. Without CNC, the acrylate emulsion can be initiated by free radicals to polyacrylate emulsion with reducing droplet size, proven by the droplet size change in Fig. 5. The addition of suitable amount of CNC can help the dispersion of acrylate and polyacrylate emulsion. After introducing excessive amount of CNC, the dispersion of acrylate droplets in emulsion might still be enhancing, proven by the droplet size, interfacial tension (Fig. 3), and zeta potential (Fig. 4). However, the stability of polyacrylate emulsion would not be as stable as the one without CNC, coagulation and aggregation happened during the polymerization and influenced the storage stability of emulsion.

### 3.3. Effect of the CNC on storage stability of polyacrylate miniemulsion

**Fig. 12:**
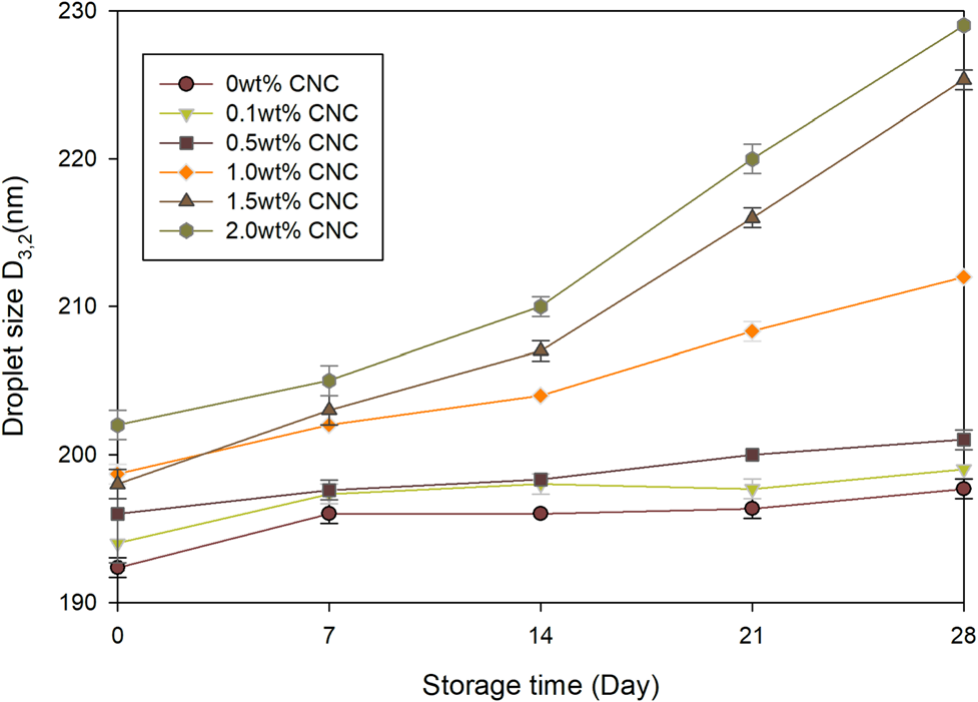
Variation in droplet size as a function of storage time.

As discussed in Section 3.2.2, the storage stability of polyacrylate miniemulsion might be influenced by the CNC contents. According to Anton and Vandamme,^59^ changes in droplet size over time could indicate the storage stability of the emulsion. To observe the effect of CNC on the storage stability of emulsion, the droplet size of acrylate miniemulsions was measured every 7 days. Although the miniemulsion appeared visually stable, droplet sizes shown in Fig. 12 increased after 4 weeks of storage at room temperature. For the 0 wt% CNC sample, the miniemulsion was relatively stable, with initial and final droplet sizes of 192 nm and 198 nm, respectively. Similarly, the samples with CNC content lower than 0.5 wt% were also stable. This might be caused by the interactions between anionic surfactants and CNC.^60^ Without CNC, the emulsion was only stabilized by SDSO with hydrophobic site in the oil phase and hydrophilic site in the water phase. After introducing CNC, it might cooperate with SDSO, where SDSO stabilized the O/W interface, and CNC formed a stable network under hydrogen bonding and electrostatic repulsion.^61,62^ However, when the content exceeded 1 wt%, CNC would apply the adverse effect on the storage stability of the miniemulsion. Especially for 2 wt% CNC sample, the droplet size increased significantly from 202 nm to 230 nm, which indicated the coagulation and aggregation of droplets.^63^ This was proven by the SEM images (Fig. 10) as a sign of deterioration in storage stability.

## 4. Conclusion

This study offers a comprehensive analysis of the interactions between CNC and PMMA-*co*-PBA in an emulsion system, both before and after polymerization. It provides valuable insights into CNC's functional roles. The results demonstrate that CNC effectively co-stabilizes acrylate miniemulsions before polymerization. While CNC did not directly participate in polymerization or affect yield or reaction rates, it slowed the diffusion of free radicals during the process. For certain concentrations, such as 1 wt%, CNC enhanced emulsion stability before polymerization but negatively impacted storage stability afterward. These findings highlight the dual role of CNC as both a stabilizing and aggregating agent, emphasizing its potential for tailoring polymer properties for industrial applications. Future research should focus on modifying CNC to improve its stabilizing effects on both the initial emulsion and the final polymerized system, while further elucidating the underlying mechanisms of its interactions with polymer matrices.

## Data availability

The data are available from the corresponding author on reasonable request.

## Conflicts of interest

There are no conflicts to declare.
